# Overcoming Detection Lag in a Cryptic Panzootic: Chytrid Invasion Linked to Historically Enigmatic Amphibian Declines in the Brazilian Atlantic Forest

**DOI:** 10.1111/mec.70573

**Published:** 2026-10-14

**Authors:** Jack L. Boyette, Tamilie Carvalho, David Rodriguez, Wesley J. Neely, Daniel Medina, Renato A. Martins, Robert J. Ossiboff, Zaira Gomez, William C. Vuyk, Shannon M. Buttimer, Karen A. Paniagua Torres, Ananda Brito de Assis, Carlos A. Navas, Carly R. Muletz‐Wolz, Michael G. Campana, Corinne L. Richards‐Zawacki, C. Guilherme Becker

**Affiliations:** ^1^ Department of Biology The Pennsylvania State University University Park Pennsylvania USA; ^2^ Center for Conservation Genomics, Smithsonian’s National Zoo and Conservation Biology Institute Smithsonian Institution Washington DC USA; ^3^ Department of Ecology and Evolutionary Biology University of Michigan Ann Arbor Michigan USA; ^4^ Institute for Global Change Biology, School for Environment and Sustainability University of Michigan Ann Arbor Michigan USA; ^5^ Department of Biology Texas State University San Marcos Texas USA; ^6^ The School for Field Studies Center for Tropical Island Biodiversity Studies Bocas del Toro Panama; ^7^ Department of Biodiversity, Aquaculture Center (CAUNESP), and CBioClima, I.B. Universidade Estadual Paulista Rio Claro SP Brazil; ^8^ Department of Comparative, Diagnostic, and Population Medicine, College of Veterinary Medicine University of Florida Gainesville Florida USA; ^9^ Department of Anthropology The Pennsylvania State University University Park Pennsylvania USA; ^10^ Departamento de Biodiversidade, Instituto de Biociências Universidade Estadual de São Paulo Rio Claro SP Brazil; ^11^ Departamento de Fisiologia, Instituto de Biociências Universidade de São Paulo São Paulo SP Brazil; ^12^ Department of Biological Sciences University of Pittsburgh Pittsburgh Pennsylvania USA; ^13^ One Health Microbiome Center, Center for Infectious Disease Dynamics, Huck Institutes of the Life Sciences The Pennsylvania State University University Park Pennsylvania USA

**Keywords:** amphibians, chytridiomycosis, infectious disease, museum collections

## Abstract

As human activity accelerates the spread of pathogens and other invasive species, reconstructing the cryptic early dynamics of undetected invasions has become a central challenge in disease ecology and invasion biology. Here, we leverage museum specimens to reconstruct the invasion of *Batrachochytrium dendrobatidis* (Bd), a fungal panzootic driving global amphibian biodiversity loss, across historically enigmatic declines in the Brazilian Atlantic Forest. Two hypotheses have been proposed to explain Bd's historical dynamics in this region: long‐term enzootic persistence versus recent epizootic invasion coinciding with declines. Using molecular screening, histopathology, and genotyping of museum and modern specimens, we find no evidence of Bd infection prior to the decline, followed by widespread infection immediately thereafter. Only the global panzootic lineage (Bd‐GPL) was detected during the first year of post‐decline sampling, implicating its invasion in driving regional losses. Infection prevalence varied among species following the decline, likely reflecting species‐specific interactions between baseline susceptibility and ecological exposure risk. Contemporary host populations exhibit more uniform prevalence regardless of decline history, consistent with the evolution of pathogen tolerance under enzootic conditions. Together, these results provide the first site‐specific evidence linking Bd‐GPL invasion to enigmatic amphibian declines in Brazil and demonstrate the power of museum collections for resolving detection lag dynamics in emerging infectious diseases.

## 1 Introduction

Human activity increasingly facilitates the global spread of invasive species at a speed that outpaces our ability to detect and respond to these biological invasions (Moody et al. 2016; Kraemer et al. 2019; Cucchi et al. 2020; Li et al. 2021; Barquera et al. 2025). This dynamic often creates detection lags that obscure our understanding of the critical early stages of invasion (Crooks 2005), especially for pathogens, which are easily transported, difficult to detect, and inherently harmful to their hosts. Consequently, many pathogen invasions must be reconstructed retrospectively (Cheng et al. 2011; Croucher et al. 2013; Vredenburg et al. 2019; Crits‐Christoph et al. 2024). Such retrospective approaches are essential for inferring the dynamics of pathogen emergence and spread, as well as understanding subsequent host responses (Strayer et al. 2006).

This challenge is exemplified by the chytrid fungus *Batrachochytrium dendrobatidis* (Bd), which has contributed to hundreds of amphibian species declines and at least 90 documented extinctions (Scheele et al. 2019). These declines peaked globally in the 1980s (Scheele et al. 2019), well before the pathogen's formal description in 1999 (Longcore et al. 1999). This chronology reveals a multi‐decade detection lag that obscures Bd's early history. The resulting uncertainty complicates estimates of the pathogen's toll on biodiversity and fuels debate over how many historical declines can be robustly attributed to Bd (Lambert et al. 2020). To overcome this gap and better reconstruct Bd's emergence, spread, and impacts, we must rely on inferential analyses of historical decline records and retrospective pathogen screening of museum‐preserved specimens (Lips et al. 2008; Rodriguez et al. 2014; Carvalho et al. 2017). While museum‐based studies face notable challenges, including uneven spatiotemporal sampling (Boakes et al. 2010; Oliveira et al. 2016), false negatives due to DNA degradation (Hykin et al. 2015; Hahn et al. 2022), and the potential for contamination in commingled specimen jars (Raxworthy and Smith 2021), they nonetheless provide rare insight into detection lag dynamics that would otherwise be lost to time.

Critically, Bd is a genetically diverse complex of lineages with varying ecological and epidemiological profiles. Most historical and contemporary declines are attributed to Bd's global panzootic lineage (Bd‐GPL), which appears to be a relatively recently diverged, globally distributed, and hypervirulent lineage (Farrer et al. 2011; O'Hanlon et al. 2018). In contrast, several more deeply diverged, geographically restricted, and typically lower virulence lineages have been described, including Bd‐Asia1 (O'Hanlon et al. 2018), Bd‐Asia2/Bd‐Brazil (hereafter Bd‐Brazil) (Schloegel et al. 2012), Bd‐Asia3 (Byrne et al. 2019), and Bd‐Cape (Farrer et al. 2011). These lineages are thought to represent long‐term enzootics in their respective regions based on their typically mild virulence towards sympatric amphibians, which likely reflect long histories of coevolution with local hosts (Becker, et al. 2017; Carvalho, et al. 2024), especially considering that many of these lineages have demonstrated the capacity for devastating virulence following introduction into naïve host populations, both in nature (e.g., Bd‐Asia1 (Fu and Waldman 2019) and Bd‐Cape (Sewell et al. 2024)) and in laboratory experiments (e.g., Bd‐Brazil (Becker, et al. 2017)). These patterns emphasize that lineage identity, beyond the simple presence of Bd, can influence whether amphibian populations persist or collapse. This distinction is important when reconstructing the timing of Bd invasion, as the presence of an enzootic lineage may obscure the arrival of a panzootic invader if no genotyping efforts are made. Still, most historical studies do not attempt Bd genotyping (Ribeiro et al. 2026), underscoring a critical oversight for any retrospective studies aiming to reconstruct local invasion histories in regions with high lineage diversity.

The Brazilian Atlantic Forest exemplifies this challenge. It is a global amphibian biodiversity hotspot where Bd's natural history is complicated by the coexistence of a putatively long‐term enzootic lineage (Bd‐Brazil), the global panzootic lineage (Bd‐GPL), and hybrids of the two (Rodriguez et al. 2014; Schloegel et al. 2012; Ribeiro et al. 2026; Jenkinson et al. 2016). Enigmatic amphibian declines in the Atlantic Forest peaked in 1979 (Toledo et al. 2023), and reconstructions of Bd's detection lag dynamics have given rise to two competing hypotheses. The enzootic pathogen hypothesis focuses on evidence of Bd's presence in the Atlantic Forest for more than a century (Rodriguez et al. 2014; Ribeiro et al. 2026). In contrast, the spreading pathogen hypothesis posits that Bd invaded more recently, coinciding with the global expansion of Bd‐GPL and the peak of regional declines (Jenkinson et al. 2016; Carvalho et al. 2017). Museum‐based studies have supported both hypotheses (see Rodriguez et al. 2014 for the enzootic pathogen hypothesis and Carvalho et al. 2017 for the spreading pathogen hypothesis), though these efforts prioritized spatial and temporal breadth over the fine, single‐site resolution necessary to capture the dynamics of specific declines.

The most extensive and best‐documented amphibian declines in the Atlantic Forest occurred between 1979 and 1982 at the Boracéia Biological Station. These declines included drastic reductions in six species and the apparent extirpation of five others, while 16 species reportedly maintained stable abundance (Heyer et al. 1988). These reports were limited to common, conspicuous species for which abundance changes could be reliably inferred. Although the authors acknowledged that their ‘field data were not collected in a form that allows precise quantification of collecting effort’, they noted that ‘special efforts’ were made to collect species perceived as decimated or extinct, resulting in relatively greater collection effort in the post‐decline period. These specimens are now housed in the Smithsonian's herpetology collection and span two pre‐decline field campaigns in the 1970s (1975 and 1976‐1977) and three post‐decline campaigns in the 1980s (1983, 1984, and 1988). Heyer et al. (1988) estimated that the decline peaked in 1979; however, no sampling occurred between 1978 and 1982 due to political instability and prohibitively high fuel costs (Toledo et al. 2023). Despite this temporal gap, the Boracéia collection remains one of the most robust amphibian collections spanning a well‐documented decline, both regionally and globally.

In hindsight, the timing of the Boracéia decline coincides with the rapid regional and global expansion of Bd‐GPL (Lips et al. 2008; Scheele et al. 2019) as well as the wave of amphibian losses reported throughout the Atlantic Forest (Weygoldt 1989; Carvalho et al. 2017; Toledo et al. 2023), collectively aligning with the spreading pathogen hypothesis. If this hypothesis is correct, we would predict an increase in Bd prevalence corresponding to the decline, likely driven by invasion of Bd‐GPL. The Boracéia collection provides a powerful opportunity to test these predictions. To do so, we apply molecular diagnostics, histopathology, and SNP‐based genotyping to a taxonomically broad series of specimens collected before and after the decline. This approach enables us to reconstruct Bd prevalence and lineage diversity across the event, providing a framework to test if cryptic Bd invasion corresponded with the Atlantic Forest's best‐documented amphibian declines. By extending sampling to modern Boracéia frogs, we further test for indirect signatures of disease tolerance evolution among species that historically declined. Finally, we outline key methodological considerations for researchers working with museum‐preserved amphibian specimens, emphasizing strategies to minimize contamination risk, account for molecular degradation, and ensure robust inference when reconstructing historical detection lag dynamics of Bd invasion.

## 2 Materials and Methods

### 2.1 Museum‐Preserved Specimen Sampling

For this study, we sampled 17 total species from the museum collection; however, only 11 species are represented across the decline due to the near or total extirpation of six species (Table S1). In total, we sampled 327 post‐metamorphic frog specimens, with catalogue numbers and associated metadata available in our Figshare dataset. Of these, 21 specimens were collected in the 1940s, 209 in the 1970s, and 97 in the 1980s. Although the initial preservation method for each specimen is undocumented, personal communication with former collections manager Steve Gotte confirmed that Heyer did not engage in any known aberrant collection or fixation protocols. Thus, all specimens are presumed to have been formalin‐fixed and have since been preserved in 70% ethanol for long‐term storage. Specimens were stored in jars segregated by species and then further separated by collection decade or year, meaning that no pre‐ and post‐decline specimens were stored together in the same jar.

Inferences about each species' decline history were based on two criteria: (1) explicit observations of post‐decline species abundance reported by Heyer et al. (1988), and (2) the number of specimens collected in each decade (Table S2). Based on this combination of qualitative and quantitative evidence, we assigned each of the 17 sampled species to one of 3 a priori decline history groups. The no‐decline group includes four species that Heyer et al. (1988) described as maintaining steady population abundance and for which more specimens were collected after the decline in the 1980s. The observed‐decline group includes three species that Heyer et al. (1988) explicitly noted as having undergone ‘drastic’ population declines and for which there were fewer specimens collected in the 1980s. The remaining four species were classified as decline‐ambiguous given mixed or incomplete evidence. Among these, *Haddadus binotatus* was not mentioned by Heyer et al. (1988) but was represented by fewer specimens in the 1980s. The other three species, *Scinax hayii* , *Boana bandeirantes* , and *Rhinella ornata* , were explicitly named as maintaining steady population abundance, yet were represented by fewer specimens in the 1980s. This discrepancy may reflect the sampling strategy described in Heyer et al. (1988), which prioritized ‘species that seemed to be decimated or extinct’. Yet more specimens were collected in the 1980s for two no‐decline species ( *Scinax crospedospilus* and *Rhinella icterica* ), suggesting that Heyer's sampling strategy did not preclude extensive collection of no‐decline species (unfortunately, most of the *R. icterica* specimens could not be located for sampling). Given this inconsistency, we treated the decline histories of *S. hayii* , *B. bandeirantes* , and *R. ornata* as decline‐ambiguous (Table S2).

To minimize the possibility of superficial contamination by free Bd zoospores or infected skin fragments suspended in commingled preservation jars, each specimen was thoroughly rinsed with molecular‐grade ethanol diluted to 70% with sterile water (Cheng et al. 2011). Following rinsing, each specimen was swabbed with a sterile rayon swab following standard Bd sampling protocols for museum‐preserved specimens (Cheng et al. 2011; Hyatt et al. 2007). Nitrile gloves were changed between each specimen to further reduce the risk of cross‐contamination. Additionally, for each jar sampled, we submerged a swab in the storage ethanol and swabbed the inner walls and rim of the jar to assess whether Bd DNA was detectable. In total, we collected 33 jar swabs using this method.

### 2.2 Museum‐Preserved DNA Extraction

Swabs were extracted using a modified 4‐day phenol‐chloroform protocol that integrates steps from two published methods optimized for DNA recovery from museum‐preserved amphibian specimens (protocol outlined in Section S1.1) (Hykin et al. 2015; O'Connell et al. 2022). All extractions were conducted at the Smithsonian National Zoo and Conservation Biology Institute's dedicated ancient DNA laboratory to minimize the risk of contamination during historical DNA processing (Cooper and Poinar 2000). A total of 364 samples were extracted across two lab campaigns: the first in Summer 2023 (*N* = 184) and the second in Fall 2024 (*N* = 180). Of these, 33 were jar swab controls and 4 were tube‐blank extraction controls (Table S1).

Because DNA cannot always be reliably extracted from museum‐preserved specimens (Hykin et al. 2015), we validated extraction success by quantifying DNA concentration in most samples using an Invitrogen Qubit 4 fluorometer and the High Sensitivity dsDNA assay kit. Given concerns that formalin fixation and long‐term ethanol preservation may promote double‐stranded DNA (dsDNA) denaturation to single‐stranded DNA (ssDNA) (Srinivasan et al. 2002), we also quantified ssDNA concentration in a subset of extracts. Both dsDNA and ssDNA were measured in 98 samples; only dsDNA was measured in 216 samples, and only ssDNA was measured in 24 samples. Beyond measuring DNA quantity, we assessed DNA quality in a subset of 111 samples by quantifying the fragment size distribution of dsDNA using an Agilent 4150 TapeStation automated electrophoresis system with High Sensitivity D1000 ScreenTapes and reagents. Average fragment size for each sample was estimated using a region analysis between 30 and 1000 bp. Because DNA extracted from museum‐preserved specimens is often highly fragmented due to chemical degradation and age (Zimmermann et al. 2008), fragment size provides an important metric of DNA integrity that is not captured by concentration estimates alone. This information is particularly relevant to PCR‐based studies, as extensive fragmentation can limit the feasibility of amplifying large target amplicons.

### 2.3 Bd Infection Assays for Museum‐Preserved Specimens

Specimen infection status was initially quantified using a probe‐based qPCR assay with primers specific to Bd's ITS1 gene region (Boyle et al. 2004), run initially in singlicate. Bd ITS1 standards were included on each plate for relative quantification. For Summer 2023 samples, we used plasmid standards (3.64–3.64 × 10^6^ ITS1 gene copies; Pisces Molecular), and for Fall 2024 samples we used synthetic gBlock standards (10–10^6^ ITS1 gene copies; Integrated DNA Technologies). Gene copy number estimates derived from plasmid or synthetic standards have been shown to be comparably sensitive and reliable (Conte et al. 2018; Xu et al. 2019; Han et al. 2023). We did not incorporate Bd load estimates in analyses because the effects of unknown fixation conditions and preservation histories on copy number estimates cannot be accounted for. Instead, we focused solely on infection status in our analysis (i.e., positive vs. undetected), with positive assays defined as those that successfully amplified before cycle 50. To screen for PCR inhibition and reduce the likelihood of false negatives, we included TaqMan Exogenous Internal Positive Control reagents (IPCs) in each reaction (Brannelly et al. 2020). All IPCs amplified successfully, indicating minimal or no inhibition. Each plate also included at least one blank qPCR control, none of which amplified. Reaction components and qPCR cycling conditions are described in Section S2.

We attempted to replicate singlicate qPCR‐based Bd infection calls using a Bio‐Rad QX600 Droplet Digital PCR System (ddPCR) with the same reagents in a subset of 84 samples. This subset included 42 qPCR positive samples, 42 qPCR undetected samples (including 8 jar swabs or tube blank extraction controls), and 7 plasmid standards. Reaction components and ddPCR cycling conditions are described in Section S3. Discrepancies between qPCR and ddPCR calls (*N* = 5) were resolved through an additional round of duplicate qPCR with synthetic standards, for a total of three assay calls per sample. Among these, samples were considered positive if two of three assays amplified.

### 2.4 Histopathology

We further validated our molecular infection assays by collecting skin tissue for histopathological examination in a subset of 55 specimens. This subset included 15 specimens with high Bd infection loads (> 900 ITS1 gene copies as estimated via qPCR), 15 with low infection loads (< 900 ITS1 gene copies), and 25 putatively Bd‐negative specimens (21 from the 1970s pre‐decline period and 4 from the 1980s post‐decline period). We collected ventral pelvic skin and, when present, we also sampled interdigital skin. We targeted these regions because they are predilect sites for Bd infection (Berger et al. 2005; Puschendorf and Bolaños 2006; Van Rooij et al. 2015). In total, we collected 50 ventral pelvic skin samples and 31 interdigital skin samples. Tissues were secured in folded embedding sponges and placed inside screw‐cap tubes containing 2 mL of ethanol. Skin samples were then transferred to cassettes, processed routinely, sectioned at 5 μm, and stained with haematoxylin and eosin (H&E) at the Histology Laboratory at the University of Florida Veterinary Hospital. All H&E slides were first scanned to ensure appropriate sectioning and orientation and then interpreted by RJO and ZG, who were blinded to each specimen's putative infection status.

Haematoxylin and Eosin (H&E) stained sections were first assessed for the presence of Bd organisms and microscopic skin lesions potentially related to Bd infection, including hyperkeratosis and epidermal hyperplasia. Inferences from the initial round of H&E staining guided the selection of 37 samples for Periodic Acid‐Schiff (PAS) staining, a common histochemical stain used to highlight fungi in tissue sections through fuchsinophilic staining of polysaccharides in fungal cell walls (Puschendorf and Bolaños 2006). The subset of samples selected for PAS stain included 15 discrepancies between molecular and H&E results, 10 clear H&E visualizations of Bd organisms consistent with molecular detection, 8 H&E non‐detections consistent with molecular non‐detections, and 4 molecular non‐detections that exhibited evidence of non‐specific skin changes on H&E that can be seen in Bd infections. Agreement between molecular and histopathological infection diagnoses was quantified using percent agreement and Cohen's kappa (κ), calculated from a 2 × 2 contingency table of binary infection calls. Cohen's kappa was calculated using the irr package (version 0.84.1) (Gamer et al. 2019) in R.

### 2.5 Modern Sample Collection, DNA Extraction, and Infection Assays

To sample modern frogs from the same locality as Heyer et al. (1988), we conducted six field sampling campaigns at the Boracéia Biological Station between October 2022 and November 2024. All sampling was completed during the rainy season. Frogs were captured during visual encounter surveys using clean plastic bags or nitrile gloves. After capture, individuals were rinsed with distilled water and swabbed with sterile rayon swabs using standard protocols for Bd sampling (Hyatt et al. 2007). Swabs were stored dry in sterile 1.5 mL microcentrifuge tubes on ice during fieldwork and subsequently transferred to a −20°C freezer until extraction. The first two field campaigns focused on sampling two species with known population declines ( *Ischnocnema henselii* and *Hylodes phyllodes* ) for a separate project, whereas the final four campaigns sampled all encountered frogs regardless of species. Across the sampling periods, we cumulatively sampled 539 frogs representing 12 of the 17 total species sampled from the museum collection. The five species we were unable to collect may still be extirpated from Boracéia or may be persisting at undetectable population abundances.

DNA was extracted from 2022 and 2023 swabs using GMax Mini Genomic DNA Kits (IBI Scientific), and from 2024 swabs using DNeasy blood and tissue kits (Qiagen). On each day of extraction, samples were selected haphazardly to minimize temporal sampling bias, and a tube blank extraction control was included to monitor contamination. Bd infection status was determined using the same probe‐based qPCR assay applied to museum‐preserved specimens (Boyle et al. 2004). Assay setup was also identical, including synthetic plasmid standards (Pisces Molecular), a TaqMan Exogenous IPC in each reaction, and a no template control (NTC) in every plate. If a reaction's IPC failed to amplify, the sample was re‐assayed. All NTCs remained negative, indicating no detectable contamination.

### 2.6 SNP‐Based Genotyping Assay

To infer Bd lineage identity in qPCR‐positive samples, we applied two SNP‐based genotyping assays to 102 swab extracts from museum and modern samples. Each assay genotypes a pair of nuclear or mitochondrial alleles based on the presence or absence of FAM/VIC probe fluorescence. This allows for identification of SNPs associated with Bd‐Brazil or Bd‐GPL. The first assay, SC9_621917 (Carvalho, Medina, et al. 2023), targets a nuclear SNP that differentiates Bd‐GPL (allele A only), Bd‐Brazil (allele C only), and either coinfection or hybrid genotypes (mixed allele A/allele C). The second assay, Bdmt_26360 (Jenkinson et al. 2018), targets a mitochondrial SNP to distinguish Bd‐GPL/hybrid (allele G only), Bd‐Brazil/hybrid (allele A only), and coinfection (mixed allele G/allele A). Because each assay provides complementary genotyping information, results were interpreted jointly to make robust lineage calls. Together, these assays enable the identification of Bd‐GPL, Bd‐Brazil, coinfection, or an F1 hybrid of both lineages (Figure S1).

### 2.7 Statistical Analyses

To test the effect of specimen age on DNA yield, we fitted Gamma‐distributed generalized linear mixed models (GLMMs) with either dsDNA or ssDNA concentration as the response variable and collection date as a fixed effect. To evaluate whether DNA concentration predicted *Bd* infection status, we fitted binomial GLMMs with infection status (positive/undetected) as the response variable, including the adjusted concentration of either dsDNA or ssDNA and a binary indicator of whether any measurable DNA was detected as fixed effects. To test whether collection date predicted DNA integrity, we fitted a Gamma‐distributed GLMM with mean fragment size as the response and collection date as a fixed effect. To assess whether fragment size predicted *Bd* infection status, we fitted a binomial GLMM with infection status as the response and mean fragment size as a fixed effect. All GLMMs included species and extraction set as random effects and were fitted using the glmmTMB package (Version 1.1.11) (Brooks et al. 2017) in R. All infection prevalence estimates are reported with 95% binomial confidence intervals. Because traditional binomial models exhibited quasi‐complete separation in *Bd* infection prevalence across zootic periods and decline history categories, we used Fisher's exact tests to compare Bd prevalence (1) between the prezootic and epizootic periods, (2–3) among a priori decline history categories in the epizootic and enzootic periods, (4) between no‐decline species and other decline history categories in 1983, and (5) between decline‐ambiguous and other decline history categories in 1988. Finally, we modelled temporal trends in *Bd* infection prevalence using generalized additive models (GAMs) with a binomial error distribution and logit link, fitted with Restricted Maximum Likelihood using the mgcv package (version 1.9–1) (Wood 2004) in R. Species‐level differences in baseline prevalence were modelled as random intercepts via a smooth term with a random‐effect basis.

## 3 Results

### 3.1 Historical Samples Show a Sharp Rise in Bd Prevalence Coincident With Decline

Using our molecular diagnostics, we detected no evidence of Bd infection among the 230 frogs collected before the 1979 decline (0% prevalence, 95% CI: 0%–1.6%; prezootic period in Figure 1). In stark contrast, 57 of the 97 post‐decline frogs collected in the 1980s tested positive for Bd (58.8% prevalence, 95% CI: 48.3%–68.7%; Table S3, epizootic period in Figure 1), revealing a significant increase in infection prevalence before versus after the 1979 Boracéia decline (Fisher's exact test, *p* < 0.001). Our GAM model supported this pattern (red line in Figure 1), with collection year exhibiting a significant nonlinear effect on infection prevalence (edf = 3.02, *χ* ^2^ = 32.14, *p* < 0.001). The species‐level random‐effect smooth was also significant (edf = 8.71, *χ* ^2^ = 33.60, *p* < 0.001), indicating substantial among‐species variation in baseline infection probability after accounting for temporal trends. The model explained 23.2% of the deviance (adjusted *R* ^2^ = 0.21; *n* = 866). No Bd was detected across all 51 controls, including 4 tube blank extraction controls, 14 qPCR plate controls, and 33 jar swab controls.

**FIGURE 1:**
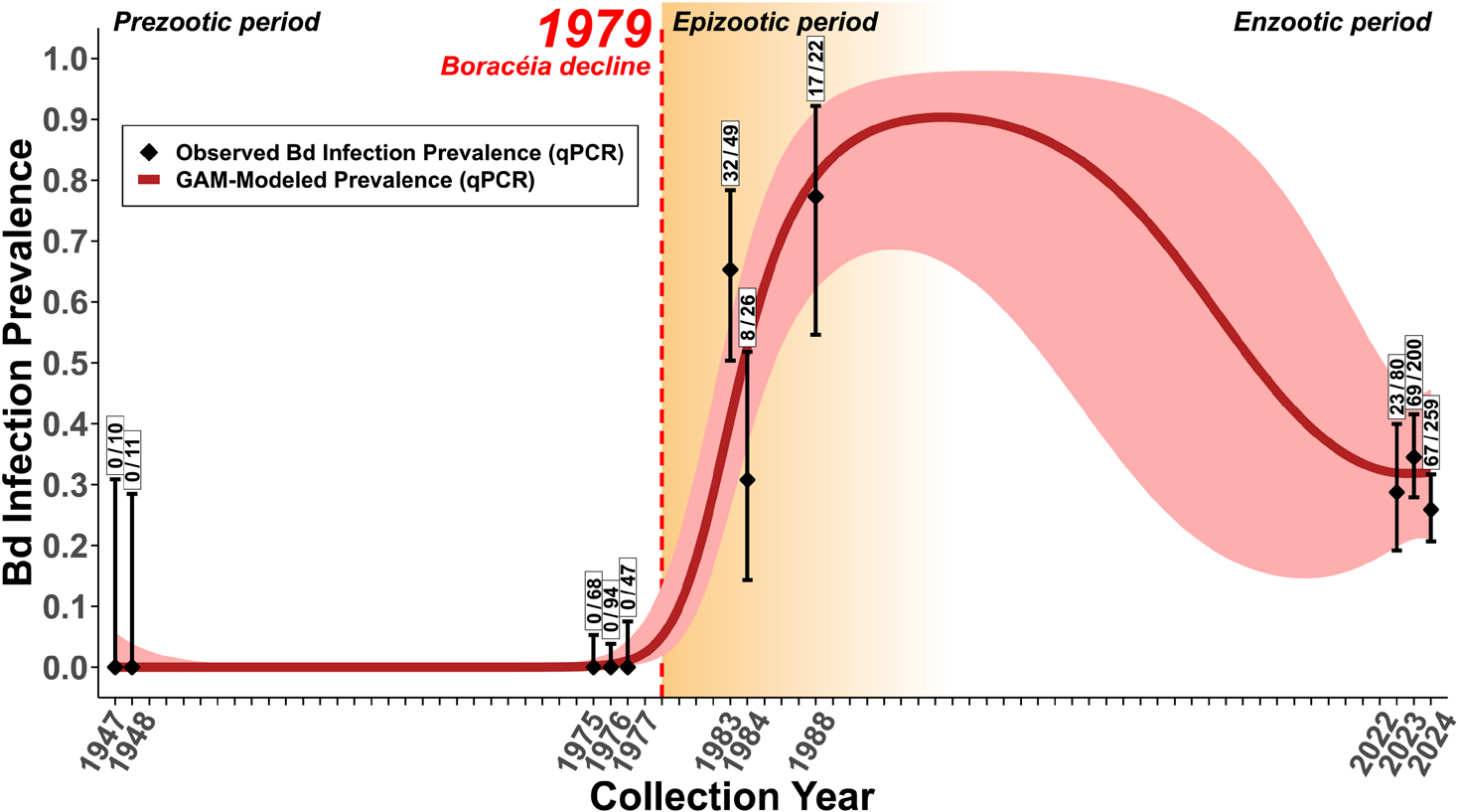
Bd infection prevalence peaked following the historical decline and later stabilized. Black diamonds represent observed annual Bd infection prevalence as estimated by qPCR with 95% binomial confidence intervals. The red line shows GAM‐predicted prevalence through time with 95% confidence intervals (red ribbon).

Distinct patterns of Bd infection were observed among the eleven species sampled in the 1980s post‐decline period, and these patterns fit the inferred decline history of each species (Figure 2). The four no‐decline species were highly infected, with species‐level prevalence ranging from 50% ( *R. icterica* ) to 100% ( *P. cuvieri* ). Collectively, Bd was detected in 81.0% of the no‐decline specimens (*n* = 34/42 infected, 95% CI: 65.9%–91.4%). Among the three observed‐decline species, Bd was detected in 0% of specimens (*n* = 0/15 infected, 95% CI: 0%–21.8%). There was no clear pattern of infection prevalence among the four decline‐ambiguous frogs, with species‐level prevalence ranging from 17% ( *H. binotatus* ) to 75% ( *R. ornata* ). Collectively, Bd was detected in 57.5% of the decline‐ambiguous specimens (*n* = 23/40 infected, 95% CI: 40.9%–73.0%). The frequency of Bd‐positives differed significantly between the three decline history categories (Fisher's exact test: *p* < 0.001).

**FIGURE 2:**
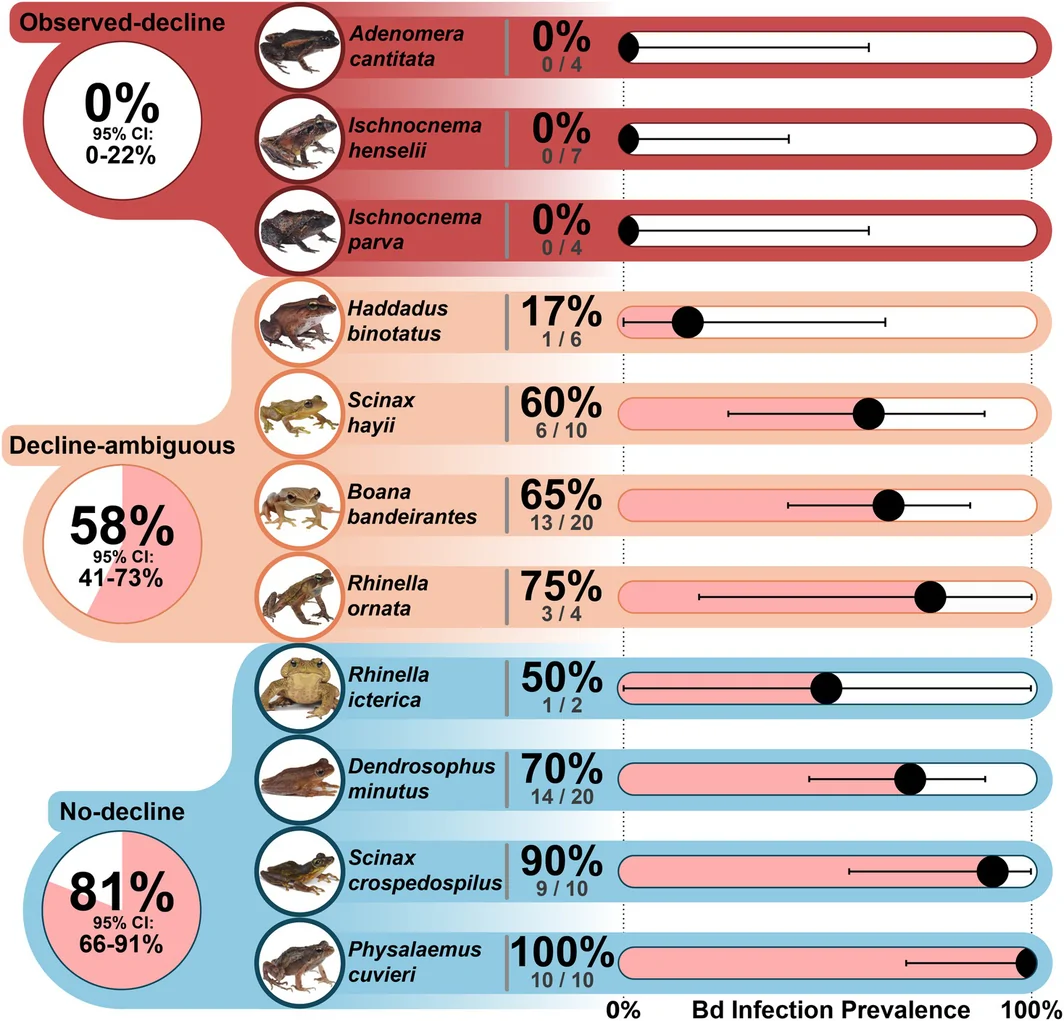
Bd infection prevalence varies among species with different decline histories. Per‐species Bd infection prevalence during the 1980s post‐decline period with 95% binomial confidence intervals. Species are grouped by a priori decline history categories, with category‐level prevalence estimates and 95% confidence intervals shown.

### 3.2 Museum DNA Degradation Does Not Explain Bd Detection Patterns

Across all museum‐preserved samples, collection date was a statistically significant predictor of dsDNA concentration (*β* = 0.527, SE = 0.180, *p* = 0.003), indicating a small increase in dsDNA quantity over time (Figure 3A). Despite this effect, neither dsDNA concentration (*β* = 0.242, SE = 0.187, *p* = 0.195) nor binary detection of dsDNA (*β* = −0.040, SE = 0.886, *p* = 0.964) was associated with Bd detection (Figure 3B). When analyses were restricted to samples collected in the 1970s and 1980s, collection date no longer predicted dsDNA concentration (*β* = 0.095, SE = 0.079, *p* = 0.230; Figure 3A), and dsDNA concentration and binary detection of dsDNA remained unassociated with Bd detection. Collection date did not predict ssDNA concentration (β = 0.099, SE = 0.213, *p* = 0.641; Figure 3C), and neither ssDNA concentration (β = −0.269, SE = 0.395, *p* = 0.496) nor binary detection of ssDNA (*β* = −0.164, SE = 1.260, *p* = 0.897) was associated with Bd detection (Figure 3D). In contrast, mean fragment size increased with collection date (*β* = 0.449, SE = 0.070, *p* < 0.001; Figure 3E) and was positively associated with Bd detection (*β* = 0.009, SE = 0.003, *p* = 0.007; Figure 3F). These relationships remained significant when analyses were restricted to 1970s–1980s samples. However, mean fragment size in 1970s specimens (232 bp, 95% CI: 191–273 bp) exceeded the ~146 bp amplicon length targeted by the Bd qPCR assay, suggesting that fragmentation was unlikely to limit Bd detection in these samples.

**FIGURE 3:**
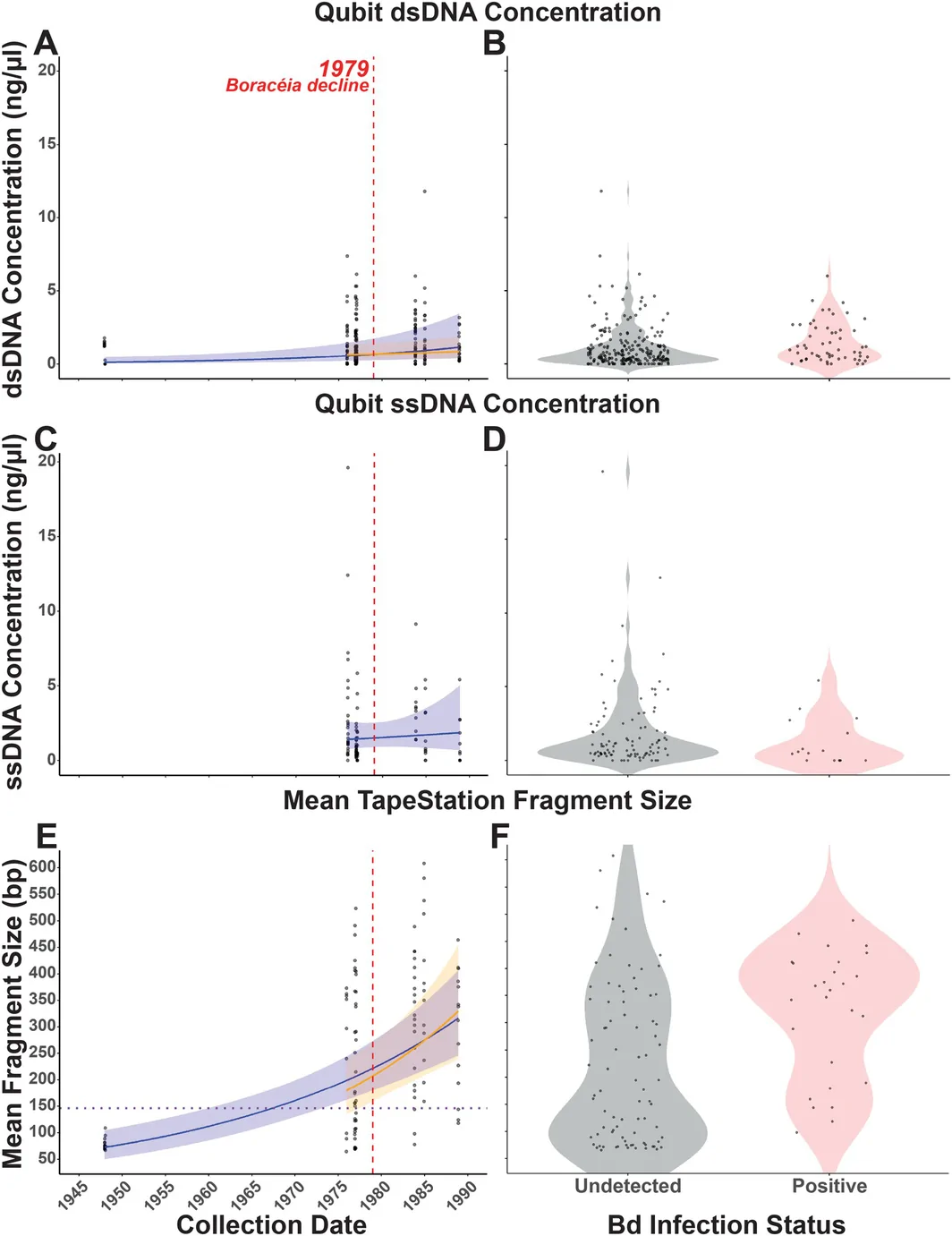
Museum DNA degradation does not explain Bd detection patterns. Models of DNA degradation through time and by Bd infection status. (A) GLMM‐modelled Qubit dsDNA concentration through time for all museum‐preserved samples (blue) and 70s/80s samples only (orange). (B) Qubit dsDNA concentration by Bd infection status. (C) GLMM‐modelled Qubit ssDNA concentration through time for all museum‐preserved samples. (D) Qubit ssDNA concentration by Bd infection status. (E) GLMM‐modelled mean TapeStation fragment size through time for all museum‐preserved samples (blue) and 70s/80s samples only (orange). Horizontal dashed line represents the amplicon size of the Bd qPCR target (~146 bp). (F) Mean TapeStation fragment size by Bd infection status.

### 3.3 Near‐Perfect Agreement Between qPCR and ddPCR Bd Detection in Museum Samples

Across 84 samples tested with both assays, qPCR and ddPCR showed high concordance (94.0% agreement; *κ* = 0.88), indicating near‐perfect agreement between methods. Among qPCR‐positive samples, 39 of 42 were replicated by ddPCR, while 40 of 42 qPCR‐undetected samples were replicated by ddPCR. The five discordant samples were nearly symmetrically distributed between assays (3 qPCR+/ddPCR−; 2 qPCR−/ddPCR+). The three qPCR positives that were not detected by ddPCR involved samples with some of the lowest loads estimated via qPCR (2.5–5.7 ITS1 gene copies as estimated by qPCR), although the ddPCR assay was able to replicate Bd detection in other samples with similarly low qPCR loads (as few as 3.8 ITS1 gene copies). To resolve the five discrepancies between our qPCR and ddPCR assays, we ran an additional set of duplicate qPCR assays using a synthetic gBlock standard. These additional assays replicated all but one of the original qPCR results, revealing one likely false positive Bd detection among the original singlicate results. All seven jar‐swab controls, one extraction control, and one no‐template control were confirmed as negative by ddPCR.

### 3.4 High Concordance Between Molecular and Histopathological Bd Detection in Museum Samples

A subset of specimens was selected for infection status validation via histopathology (Figure 4), including 15 with high loads (> 900 ITS1 gene copies as estimated by qPCR), 15 with low loads (< 900 ITS1 gene copies), and 25 without Bd detection. Overall, molecular and histopathological diagnoses showed high concordance (85.5% agreement; *κ* = 0.71), indicating substantial agreement between methods. Bd sporangia were visualized in 93.3% (*n* = 14/15) of high load specimens. The single high load specimen in which Bd was not observed showed no evidence of skin pathology and had the lowest qPCR load estimate within this group (934 ITS1 copies; USNM 303015). Among low‐load specimens, Bd sporangia were visualized in 53.3% (*n* = 8/15) samples. No Bd organisms were observed in any of the putatively Bd‐negative specimens (*n* = 0/25), including 21 specimens collected before the 1979 decline (Figure S2).

**FIGURE 4:**
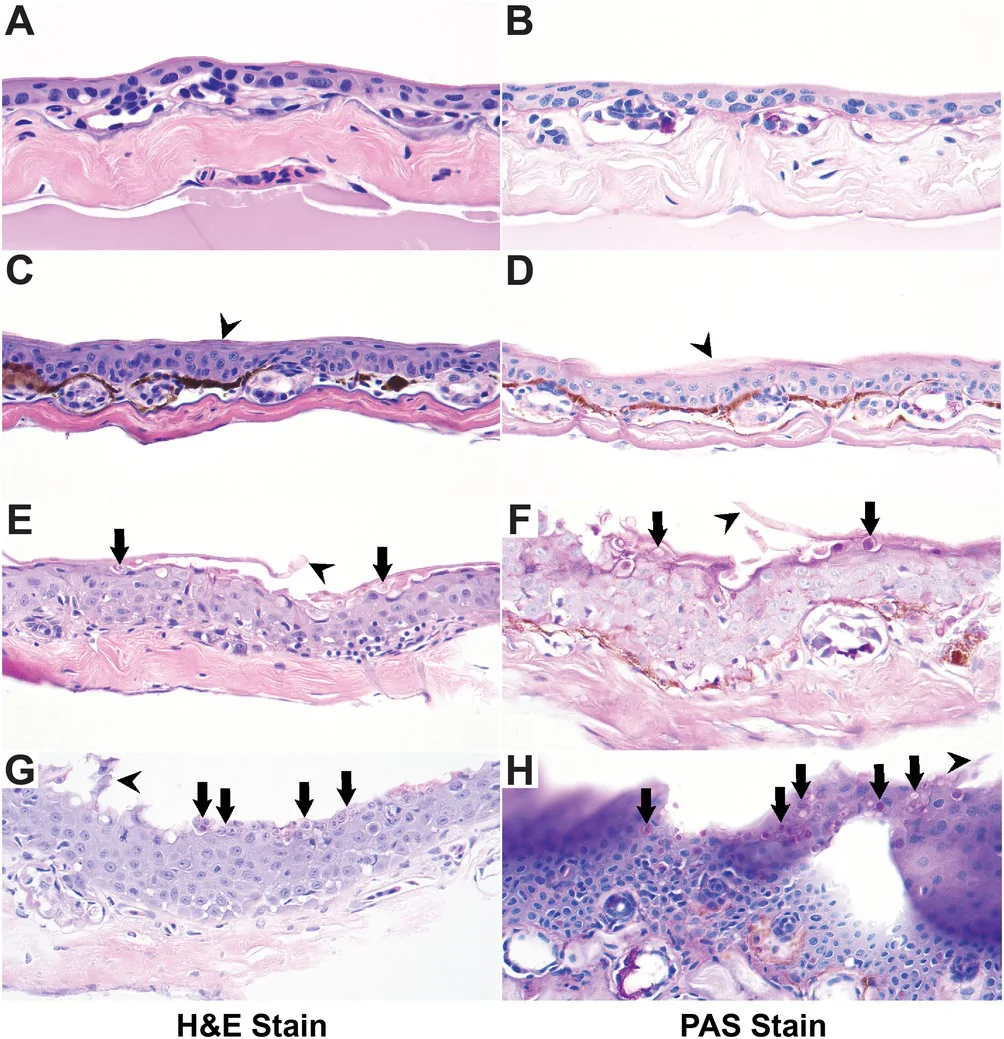
Histopathology confirms Bd infection in museum specimens. Paired photomicrographs from representative specimens examined histologically. Left panels (A, C, E, G): Haematoxylin and eosin (H&E); right panels (B, D, F, H): Periodic acid–Schiff (PAS). Arrows indicate a subset of Bd sporangia present in each section; arrowheads highlight hyperkeratosis, a non‐specific skin change in amphibians that is typical of chytridiomycosis. (A, B) USNM catalogue number 209132, characterized by histologically normal skin and no Bd sporangia. (C, D) USNM catalogue number 209080, characterized by mild epidermal hyperplasia and hyperkeratosis, but no apparent Bd sporangia. (E, F) USNM catalogue number 243622, characterized by moderate epidermal hyperplasia and hyperkeratosis, with low Bd load microscopically. (G, H) USNM catalogue number 243579, characterized by marked epidermal hyperplasia and hyperkeratosis and moderate numbers of Bd sporangia microscopically. All photomicrographs are of ventral pelvic skin samples taken at 400× magnification.

### 3.5 Contemporary Bd Prevalence Is Reduced and No Longer Varies by Decline History

Among 539 swabs collected from modern Boracéia frogs, 159 were Bd‐positives by qPCR (29.5% prevalence, 95% CI: 25.7%–33.5%; Table S4; enzootic period in Figure 1). Annual prevalence remained stable across the three sampling years (25.9%–34.5%). In contrast to patterns observed in the post‐decline period, contemporary prevalence did not differ among decline history categories (Fisher's exact test; *p* = 0.686; Figure S3). No‐decline species had an overall infection prevalence of 30.4% (*n* = 21/69 infected, 95% CI: 19.9%–42.7%), compared with 30.1% among observed‐decline species (*n* = 120/398 infected, 95% CI: 25.7%–34.9%), and 25.0% among decline‐ambiguous species (*n* = 18/72 infected, 95% CI: 15.5%–36.6%).

### 3.6 Genotyping First Identifies Bd‐GPL Following Declines With Subsequent Bd‐Brazil and Hybrid Detections

Overall genotyping efficiency in this study, defined as the proportion of assays that successfully amplified before cycle 50, was 47.5% across all samples (*n* = 97/204). Genotyping efficiency was similar between assays and decades, although the interaction between assay and decade was significant (binomial GLM; assay: *χ* ^2^_1_ = 1.59, *p* = 0.207; decade: *χ* ^2^_1_ = 0.04, *p* = 0.833; assay × decade: *χ* ^2^_1_ = 3.88, *p* = 0.049). Focusing on successfully genotyped SNPs (*n* = 194/408), SNP identity did not differ across decades (Pearson's *χ* ^2^_2_ = 0.44, *p* = 0.802). Nuclear assays amplified less frequently in modern samples, resulting in a greater number of incomplete consensus lineage calls because hybrid identity cannot be ruled out when only allele G or allele A mitochondrial SNPs are genotyped. Despite this, there were more hybrid consensus lineage calls in modern samples, and the only detected coinfection (Figure 5B). In the first sampling period following the declines (1983), all successfully genotyped SNPs (Figure 5A) and all consensus lineage calls (Figure 5B) were attributed to Bd‐GPL. In the subsequent sampling period (1984), two Bd‐Brazil SNPs were genotyped from the mitochondrial assay of a single specimen, but the nuclear assay did not amplify, precluding a consensus lineage call. By 1988, Bd‐Brazil dominated both successfully genotyped SNPs and consensus lineage calls, and the first hybrid lineage was detected (Figure 5B).

**FIGURE 5:**
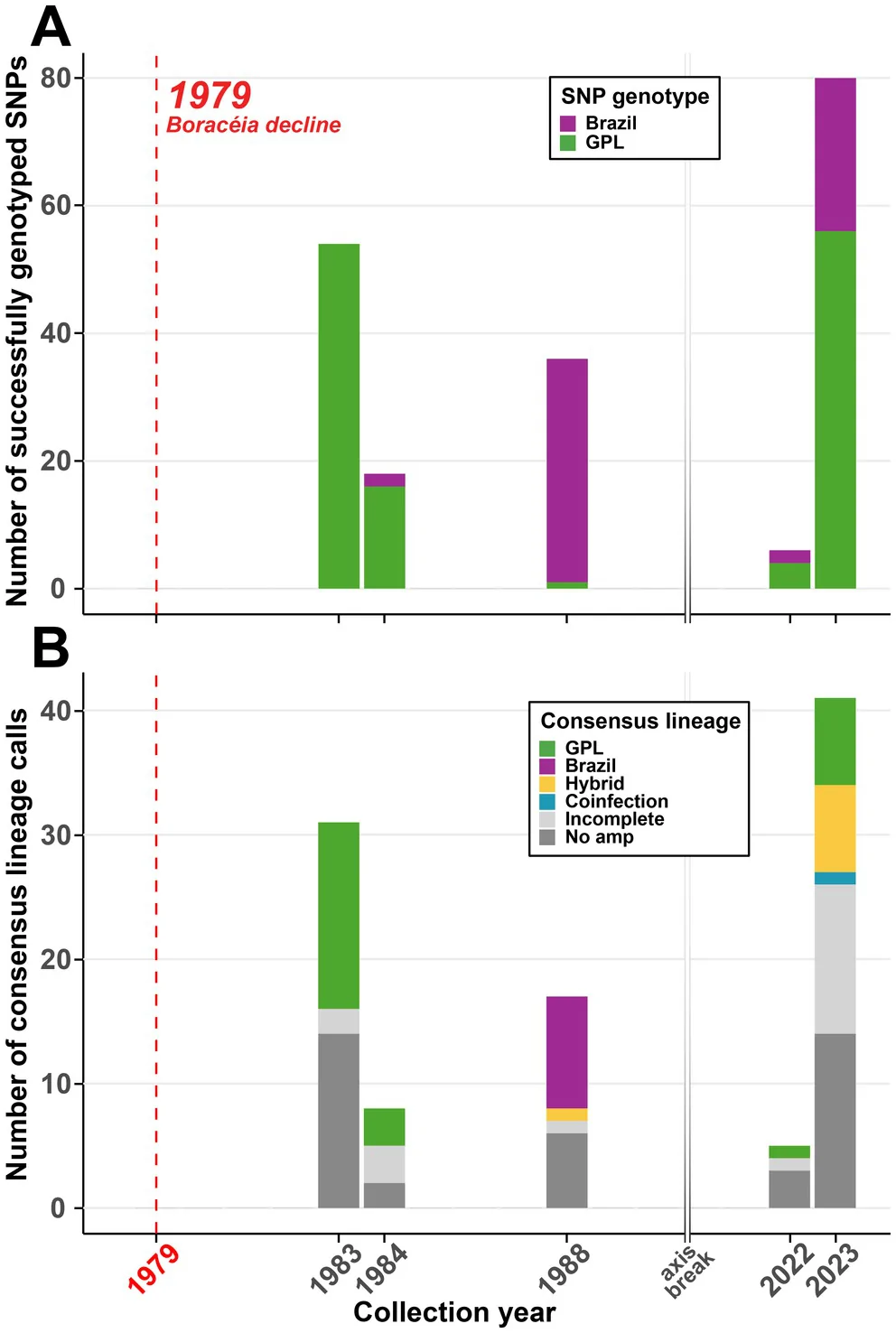
Bd genotype and lineage composition shifts through time. Genotyping results across a subset of 1980s post‐decline and modern samples. (A) Number of successfully genotyped nuclear and mitochondrial SNPs and associated Bd lineage assignments across years. (B) Number of consensus lineage calls integrating nuclear and mitochondrial SNPS within the same sample. In most cases, lineage calls were considered incomplete when only a single SNP was genotyped (see Figure S1).

## 4 Discussion

### 4.1 Boracéia Amphibian Declines Linked to Epizootic Bd

The amphibian declines observed at the Boracéia Biological Station between 1979 and 1982 are likely the best documented in the Brazilian Atlantic Forest. However, their underlying cause remains controversial (Rodriguez et al. 2014; Carvalho et al. 2017; Toledo et al. 2023), and it was recently proposed that they are better explained by climate change and extreme weather events than by Bd invasion (Ferrante et al. 2025). We do not view these explanations as mutually exclusive, as environmental conditions are known to influence Bd transmission (Moura‐Campos et al. 2021) and host susceptibility (Buttimer et al. 2024; Carvalho, Medina, et al. 2024) in the Atlantic Forest. Here, however, we focus specifically on Bd invasion and reveal a striking temporal pattern of Bd infection prevalence among frogs collected across the decline: Bd was detected in 0% of frogs collected before 1979, compared with 58.8% of frogs collected during the 1980s post‐decline period (hereafter, the epizootic period). Our molecular results are independently validated by histopathology, with no Bd organisms observed in frogs collected before 1979. While previous work identified a spatio‐temporal association between increasing Bd prevalence and amphibian declines at broader spatial scales in the Atlantic Forest (Carvalho et al. 2017), our study provides the first direct, site‐specific evidence linking Bd emergence to historically enigmatic amphibian declines in the region. These findings support the spreading pathogen hypothesis and are consistent with global patterns of Bd‐associated amphibian declines, which intensified in the late 1970s and peaked in the mid‐1980s (Scheele et al. 2019). In Latin America, independent Bd introductions have been inferred in Ecuador in 1974 (Jervis et al. 2026), Venezuela in 1977 (Lips et al. 2008), and Costa Rica during the late 1980s (Cheng et al. 2011; Lips et al. 2008). These regional introductions and associated declines parallel the timeline of declines in the Atlantic Forest, which peaked between 1979 and 1987 (Toledo et al. 2023). Together, these findings place the Boracéia declines within the broader temporal and spatial context of Bd‐associated amphibian collapses and strongly support the conclusion that they were driven by an epizootic outbreak of Bd.

Consistent with this hypothesis, we observed distinct patterns of Bd infection prevalence between species with contrasting decline histories, specifically for specimens collected in the 1980s epizootic period. Across the decade, cumulative infection prevalence was 0% among observed‐decline species, compared to 81% among no‐decline species (Figure 2). Although this pattern may appear counterintuitive, we propose two non‐mutually exclusive processes that could generate it. One possibility is that the uninfected observed‐decline frogs represent individuals that survived the epizootic bottleneck due to complete or adaptive resistance to Bd, defined as the ability to prevent or clear infection following exposure (Grogan et al. 2023). Alternatively, these frogs may represent susceptible individuals that experienced minimal exposure to Bd at the species level. Because exposure risk can confound measures of resistance in modern frogs (Grogan et al. 2023); distinguishing between these processes using our historic prevalence data alone is likely impossible. Nevertheless, differences in species ecology provide clues as to which process may be most important. The three observed‐decline species collected across the decline are all terrestrial breeders that lack an aquatic larval stage: *Ischnocnema henselii* and *I. parva* are direct developers, while *Adenomera cantitata* is a terrestrial foam nester (Haddad et al. 2013). Because these species do not rely on water to reproduce, they likely experienced limited exposure to Bd in streams or ponds, which are key environmental pathogen reservoirs (Becker et al. 2019; Buttimer et al. 2025). In contrast, the six observed‐decline species that Heyer was unable to collect in the epizootic period are all stream‐associated aquatic‐breeding taxa that likely experienced chronic Bd exposure. In effect, the only observed‐decline species persisting at abundances sufficient for collection were those with the lowest exposure risk. Supporting this interpretation, field surveys of modern Atlantic Forest amphibians indicate that direct‐developing species experience reduced Bd exposure across ontogeny, while laboratory experiments suggest that these same species exhibit little capacity for resistance following Bd exposure (Mesquita et al. 2017). Together, these findings suggest that uninfected observed‐decline frogs likely represent susceptible but minimally exposed individuals, whereas the highly infected no‐decline species likely possessed baseline tolerance to infection. Decline‐ambiguous species exhibited intermediate cumulative prevalence across the epizootic period (Figure 2), potentially consistent with intermediate levels of tolerance.

Comparing Bd prevalence by decline history categories across years provides further insight into the temporal dynamics of the epizootic (Tables S5–S7). In 1983, the first year of epizootic sampling, prevalence among no‐decline species was 92.9% (95% CI: 76.5%–99.1%), compared with 42.9% among decline‐ambiguous species (95% CI: 17.7%–71.1%; Table S7). No‐decline species were disproportionately represented among Bd‐positive specimens in 1983, with no‐decline specimens having 29.3‐fold higher odds of Bd infection than specimens from other decline history categories (Fisher's exact test, OR = 29.3, 95% CI: 5.0%–329.5%, *p* < 0.001). We speculate that the high prevalence among these taxa may have contributed to Bd persistence in the early epizootic period, potentially providing a reservoir from which infection could spill over to more susceptible species (Scheele et al. 2017). Conversely, the complete absence of Bd‐positive observed‐decline specimens, despite the documented declines of these species, is consistent with rapid mortality following infection and a consequently limited window for transmission in these taxa. Thus, our earliest epizootic samples reveal evidence of Bd infection across all decline histories, suggesting rapid community‐wide invasion in the epizootic period. In 1984, an apparent dip in annual cumulative prevalence (Figure 1) is also evident among both no‐decline and decline‐ambiguous species (Table S7), suggesting that the dip was not solely an artefact of the relatively high proportion of uniformly Bd‐negative observed‐decline specimens sampled that year (26.9%; Table S6). By 1988, prevalence among no‐decline species was 66.7% (95% CI: 22.3%–95.7%), compared with 86.7% among decline‐ambiguous species (95% CI: 59.5%–98.3%; Table S7). This difference was not statistically significant (Fisher's exact test, *p* = 0.274), in contrast to the stark difference in prevalence observed in 1983. The convergence in prevalence across no‐decline and decline‐ambiguous species suggests a late‐epizootic shift in disease dynamics towards the more evenly distributed infection prevalence observed in our modern samples, although interpretive caution is warranted given the small sample sizes in 1988. Prevalence data alone cannot resolve the mechanisms underlying interspecific differences in host susceptibility, tolerance, or competence; however, these temporal shifts in the distribution of Bd infection provide insight into the epidemiological dynamics of the epizootic and raise the possibility that hosts with contrasting decline histories played different roles in the emergence and persistence of infection. More broadly, these findings underscore the importance of community‐wide sampling when reconstructing cryptic Bd invasions, as focusing exclusively on observed‐decline species would have failed to capture the epizootic signal and yielded zero historical Bd detections in our dataset.

In our modern samples, no differences in Bd infection prevalence were observed between species with contrasting decline histories (Figure S3). Instead, all three groups exhibited prevalence near the three‐year community average of 29.5%. This pattern suggests that, despite distinct infection dynamics in the epizootic period, species across all decline history categories have converged on Bd tolerance over the subsequent four decades. Although caution is warranted when inferring tolerance or resistance phenotypes from field prevalence data in the absence of controlled infection experiments (Grogan et al. 2023), this pattern is consistent with classical theory predicting that tolerance traits should increase in frequency as infection prevalence increases (Roy and Kirchner 2000). Such selection is expected to drive tolerance homogeneity, ultimately promoting population persistence and community stability in host‐pathogen systems characterized by enzootic infection dynamics (Roy and Kirchner 2000; Råberg et al. 2008; Best et al. 2008).

### 4.2 Boracéia Epizootic Driven by Bd‐GPL Invasion

Because all genotyped SNPs in the earliest epizootic sampling year (1983) are associated with Bd‐GPL, our results suggest that Bd‐GPL invaded Boracéia between 1979 and 1982. This is consistent with the timing and dynamics of the lineage's global expansion (O'Hanlon et al. 2018; Scheele et al. 2019) and supports Bd‐GPL as the driver of the local declines and extirpations at Boracéia. In contrast, Bd‐Brazil was first detected in 1984, when lineage‐associated SNPs were genotyped from a single specimen with an otherwise incomplete consensus lineage call, suggesting a secondary invasion of Bd‐Brazil following the initial Bd‐GPL epizootic. By the next sampling period in 1988, however, Bd‐Brazil dominated both genotyped SNPs and consensus lineage calls, and we also detected the first evidence of lineage hybridization at Boracéia. Although laboratory studies suggest that Bd‐Brazil is outcompeted by Bd‐GPL in coinfections (Jenkinson et al. 2018; Carvalho, Medina, et al. 2023), possibly explaining the modern dominance of Bd‐GPL and hybrids at Boracéia, the relatively high prevalence of Bd‐Brazil in 1988 suggests that it can act as a strong competitor under certain epidemiological or environmental conditions, or within host communities already filtered by Bd‐GPL–driven declines.

While Bd infection prevalence remained high in 1988, our prevalence model predicts that this period marked the beginning of a decline towards the more stable enzootic state observed at Boracéia today. This transition coincides with the increased prevalence of Bd‐Brazil, a lineage associated with reduced virulence relative to Bd‐GPL (Becker, et al. 2017). This pattern suggests the rise of the lower‐virulence Bd‐Brazil may have contributed to broader epidemiological shifts, including changes in host community structure, susceptibility, and host–pathogen coevolution. In our modern samples, multiple Bd lineages coexist without evidence of ongoing epizootic declines, consistent with a long‐term enzootic system shaped by host filtering (Buttimer et al. 2026), pathogen competition (Carvalho, Medina, et al. 2023; Jenkinson et al. 2018), and coevolutionary dynamics (Carvalho, et al. 2024). Despite the apparently stable enzootic host–pathogen dynamics observed at Boracéia today, this multi‐lineage context warrants attention because co‐occurrence increases opportunities for coinfection and recombination, potentially making disease outcomes more context dependent and difficult to predict (Byrne et al. 2019; Carvalho, Medina, et al. 2023). The increasing frequency of hybrid genotypes is particularly concerning, as experimental studies have shown that Brazilian Bd hybrids can exhibit higher virulence than parental lineages (Greenspan et al. 2018; Carvalho, Si, et al. 2023), with important implications for transmission dynamics and long‐term disease outcomes.

### 4.3 Robust Historical Inferences Are Possible With Extensive Controls, Quantification of Degradation, and Multiple Validations

Cross‐contamination is a major concern for any museum‐based molecular study, and this possibility is especially relevant for amphibian specimens stored in commingled jars (Raxworthy and Smith 2021). Reassuringly, Bd was not detected in any of our 33 jar swab controls. To our knowledge, this study represents the first museum survey of Bd prevalence to incorporate such controls, and we recommend the routine inclusion of jar swab controls in future work. Another concern is that extensive DNA degradation in museum‐preserved specimens can inhibit PCR‐based assays (Díaz‐Viloria et al. 2005; Zimmermann et al. 2008). In our dataset, both DNA concentration and fragment size declined with specimen age. Although this degradation did not appear to impair qPCR performance in our study, likely owing to the small amplicon target size (~146 bp (Boyle et al. 2004)), we recommend that future museum studies quantify the integrity of historical DNA extracts to evaluate the risk of false negatives.

Validation with multiple, independent approaches remains critical. In our molecular screening, we initially ran singlicate qPCR assays and subsequently validated a subset of samples using singlicate ddPCR assays, which should offer improved inhibitor tolerance (Taylor et al. 2017). In practice, ddPCR did not improve detection sensitivity, and therefore we instead advocate for triplicate qPCR across all samples as a more time‐ and cost‐efficient strategy. Histopathology provides a means to independently validate molecular diagnostics. We performed histopathology using both standard haematoxylin and eosin (H&E) staining and a fungal‐specific histochemical stain (PAS). The PAS staining revealed that five preliminary H&E‐based diagnoses were likely false positives, with cross sections of duct openings and suspected intercellular edema initially misinterpreted as Bd organisms. Conversely, PAS staining also detected six low‐intensity infections that were missed during initial H&E screening. These results underscore the importance of fungal‐specific staining protocols for minimizing both false positives and false negatives, and we therefore recommend their routine use. We further recommend prioritizing histopathological screening of qPCR negative specimens and high‐intensity (> 900 ITS copy) positives, as validation of low‐intensity positives is constrained by the limited tissue area examined (Hyatt et al. 2007; Boyle et al. 2004). Although histopathology will never be as scalable as qPCR, it can reveal visible disease pathology, unambiguously confirm that qPCR positives reflect true infection rather than contamination, and detect Bd infections that escape PCR detection due to DNA degradation. With extensive contamination controls, precautions for DNA degradation, and multiple validation approaches, the forensic power of museum collections can be harnessed to recover detection lag dynamics otherwise lost to time.

## 5 Conclusion

Brazil's Atlantic Forest is a global hotspot of amphibian biodiversity and losses (Toledo et al. 2023; Eterovick et al. 2005). Yet a decades‐long detection lag has obscured the role of Bd, and some have recently gone so far as to argue that Bd‐driven declines in the Atlantic Forest are “implausible” (Ferrante et al. 2025). Our results directly challenge this view, showing that invasion of Bd coincided with and likely drove the most extensive amphibian declines documented in the Atlantic Forest. By focusing exclusively on frogs collected at Boracéia, we cannot extrapolate our findings to the broader Atlantic Forest, but our results do support the hypothesis that Bd‐GPL invasion at Boracéia triggered catastrophic die‐offs (i.e., the spreading pathogen hypothesis). Our findings indicate that while Bd may have a deep enzootic history in some regions of the Atlantic Forest (Rodriguez et al. 2014), focusing on a single site can grant the resolution necessary to capture epizootic signals that may be dilute at broader spatial scales. Resolving these detection lag dynamics is only possible through museum collections, but such studies must integrate multiple validation approaches to ensure robust inference. Overall, we demonstrate that when retrospective screening is conducted rigorously, Bd emerges as a clear driver of these historically enigmatic amphibian declines. Furthermore, despite Bd's continued presence in the region and the increased prevalence of hybrid genotypes, modern infection patterns indirectly suggest evolution of Bd tolerance among species that historically declined, possibly promoting population resilience and community stability in a system now characterized by enzootic infection dynamics.

## Author Contributions

J.L.B. conceptualized the study. J.L.B., T.C.O., and D.M. performed museum sampling. J.L.B., W.J.N., R.A.M., and S.M.B. conducted modern fieldwork. J.L.B., W.J.N., S.M.B., and K.A.P.T. performed laboratory work. R.J.O. and Z.G. conducted histopathology. D.R., C.M‐Z., M.G.C., C.R‐Z., and C.G.B. provided laboratory and logistical support and supervision. A.B.A., C.A.N., and C.G.B. secured permits for modern fieldwork. J.L.B. curated and analysed the data and wrote the original manuscript draft. All authors contributed to the review and editing of subsequent drafts.

## Funding

Research funding was provided by National Science Foundation grants to CGB (DEB‐2227340, DEB‐2413542, BII‐2120084), CRMW (IOS‐2131060), and a Smithsonian Institution Fellowship Program award to JLB. This material is based upon work supported by the National Science Foundation Graduate Research Fellowship Program under Grant No. DGE‐1255832. Any opinions, findings, and conclusions or recommendations expressed in this material are those of the authors and do not necessarily reflect the views of the National Science Foundation.

## Ethics Statement

Field sampling of amphibians was authorized by SISBIO‐Brazil (#63917; #74576; #79188; #90194), SISGEN‐Brazil (R3B282B, AE285E3) and Fundação Florestal do Estado de São Paulo (003838/2020–26; 53,900/2022–68; 0000047308/2023; 000000006533/2023). All animal handling and sample collection procedures were conducted under approval of the Pennsylvania State University's IACUC (#PROTO202102113, #PROTO202102116), the University of Alabama's IACUC (#20‐05‐3622, #21‐06‐4741), the São Paulo State University's CEUA (#1/2020, #08/2021, #10/2023), and the University of São Paulo's CEUA (#398/2022).

## Conflicts of Interest

The authors declare no conflicts of interest.

## Supporting information


**Table S1:** Summary of all extracted swabs by species, decline‐history category, and decade, with summary of extracted control swabs below.
**Table S2:** Evidence supporting decline history inferences for each species.
**Table S3:** Bd prevalence and binomial 95% confidence intervals (95% CI) for each species sampled at Boracéia during the 1980s post‐decline period.
**Table S4:** Bd prevalence and binomial 95% confidence intervals (95% CI) for each species sampled at Boracéia during the 2020s.
**Table S5:** Proportion of specimens in each decline history category by year in the pre‐decline period.
**Table S6:** Proportion of specimens in each decline history category by year in the post‐decline period.
**Table S7:** Proportion of Bd‐positive specimens from each decline history category by year during the post‐decline period.
**Figure S1:** Predicted Bd consensus lineage based on combined genotyping results from the nuclear and mitochondrial SNP assays. Boxes are coloured based on the lineage associated with each lineage (blue: Bd‐GPL, teal: coinfection, green: hybrid, yellow: Bd‐Brazil). A black outline indicates that a call can be made using only the mitochondrial assay result.
**Figure S2:** Consensus histopathology results across the 1970s and 1980s. These results integrate pathological diagnostics from both H&E stain and fungal‐specific PAS stain. Dark red line represents GAM‐modelled predictions for the proportion of samples with detectable Bd sporangia across years with a 95% confidence interval ribbon. Vertical dashed red line represents the observed 1979 Boracéia decline.
**Figure S3:** Bd infection prevalence is reduced and no longer varies by decline history during the 2020s enzootic period. Per‐species Bd infection prevalence is shown with 95% binomial confidence intervals. Species are grouped by a priori decline history categories, with category‐level prevalence estimates and 95% confidence intervals shown.

## Data Availability

The data that support the findings of this study are openly available in figshare at https://doi.org/10.6084/m9.figshare.31272358.
