# Supplementary material for: Overcoming Detection Lag in a Cryptic Panzootic: Chytrid Invasion Linked to Historically Enigmatic Amphibian Declines in the Brazilian Atlantic Forest
Source: Mol Ecol. 2026 Oct 14;35(19):e70573. doi: 10.1111/mec.70573 (PMC13662623; doi:10.1111/mec.70573)
Supplement: Supplementary file 1 [file MEC-35-e70573-s001.docx]

**Supporting Information**

Overcoming detection lag in a cryptic panzootic: chytrid invasion linked to historically enigmatic amphibian declines in the Brazilian Atlantic Forest

Jack L. Boyette, Tamilie Carvalho, David Rodriguez, Wesley J. Neely, Daniel Medina, Renato A. Martins, Robert J. Ossiboff, Zaira Gomez, William C. Vuyk, Shannon M. Buttimer, Karen A. Paniagua Torres, Ananda Brito de Assis, Carlos A. Navas, Carly R. Muletz-Wolz, Michael G. Campana, Corinne L. Richards-Zawacki, C. Guilherme Becker

**Table of Contents**

Supplementary Methods	3 – 9

Section 1. Formalin-fixed DNA Extraction Protocol for Swabs	3

Section 2. qPCR reaction components and cycling conditions	8

Section 3. ddPCR reaction components and cycling conditions	9

Supplementary Tables	10 – 13

Table S1	10

Table S2	11

Table S3	12

Table S4	12

Table S5	13

Table S6	13

Table S7	13

Supplementary Figures	14 – 16

Fig. S1	14

Fig. S2	15

Fig. S3	16

**Supplementary Methods**

*Section 1. Formalin-fixed DNA Extraction Protocol for Swabs*

Protocol adapted from: Michael Yuan, Rayna Bell, Kyle O’Connell, Sarah Hykin,

Allie Byrne (6/21/21), Umilaela Arifin + Lydia Smith (1/3/23), Jack L. Boyette (10/9/2024)

DNA Lysis:

- vortex, centrifuge, heat block, thermoshaker or oven with rotator

- 1.5 ml locking tubes

- sterile plastic pestle and/or scissors (don’t need for swabs)

- cell lysis buffer (homemade: 100 mM NaCl, 100 mM Tris HCl pH 8, 25 mM EDTA pH 8, 0.5% SDS)

[For 50 mL :

- 1 mL of 5M NaCl
- 5 mL of 1M Tris HCl pH 8
- 2.5 mL of 500 mM EDTA pH 8
- 2.5 mL of 10% SDS,
- 39mL of water (or fill to 50 mL line of the Falcon tube)]

- proteinase K (20 mg/mL)

DNA Extraction:

- 25:24:1 phenol:chloroform:isoamyl alcohol

- chloroform

- 100% isopropanol

- 3M sodium acetate, pH 5.0-5.2

- glycogen (20 mg/µl)

- freshly prepared 85% ethanol

- Elution Buffer (10 mM Tris-HCl, pH 8.0)

- 0.5 mL 1 M Tris-HCl + 49.5 mL water

**Day 1: approximate time for 24 samples - 1 hour (TIPS: 200 or 1,000 μl)**

1) If swabs are stored in ethanol allow the swabs to dry out overnight by leaving them in a clean hood with the cap open. It is good to remove as much ethanol as possible to ensure they dry all the way. If swabs are stored dry, proceed to Day 2 protocol steps.

**Day 2: approximate time for 24 samples – 1 hour (TIPS: 200 μl and 1,000 μl)**

Before beginning the Day 2 protocol steps:

- Check stock of Cell Lysis Buffer and prepare more if necessary
- Aliquot Proteinase K
  - 12 samples: ~745 μl
  - 24 samples: ~1,490 μl

2) Add the following directly to the tube with the dry swab.

- 300 µl Cell Lysis Buffer
- 60 µl Proteinase K

3) Vortex all tubes for 20s and pulse spin down.

4) Incubate all samples at 55C for 12-24 hrs in the rotator inside the oven.

**Day 3: approximate time for 24 samples – 4 hours (TIPS: 200 μl, 1,000 μl, and 5 mL)**

Before beginning the Day 3 protocol steps:

- TIPS: need 5 mL, 1000 μl, and 200 μl
- Set glycogen out to thaw
- Pre-heat a thermomixer or heat block to 70C
- In a fume hood (Make sure hood is set to EXTRACTING – NOT RECIRCULATING) aliquot 25:24:1 phenol:chloroform:isoamyl alcohol (stored in fridge) into falcon tube (# of samples x 376μl) and bring to room temperature
  - 12 samples: phenol ~2,300 µL, chlorofrm. ~2,200 µL, isoamyl ~100 µL
  - 24 samples: phenol ~4,600 µL, chlorofrm. ~4,400 µL, isoamyl ~200 µL
- Make a precipitation premix by pipetting and mixing the following components (volumes are given per sample and already contain padding for pipetting error):
  - 220 µL elution buffer (10 mM Tris-HCl) (x12 = 2,640 µL, x24 = 5,280 µL)
  - 55 µL 3M sodium acetate (x12 = 660 µL, x24 = 1,320 µL)
  - 2.75 µL glycogen (x12 = 33 µL, x24 = 66 µL)
- Aliquot chloroform:
  - 12 samples: 4,560 µL
  - 24 samples: 9,120 µL
- Aliquot isopropanol:
  - 12 samples: 6,000 µL
  - 24 samples: 12,000 µL

5) When the heat block is at 70C, remove the sample tubes from the incubator. Pulse spin to collect all condensation. Then heat sample tubes to 70C for 90 min in heat block. Use a piece of foil to cover the top of the block if there is no lid.

6) Label 3 sets of 1.5 mL tubes for each sample (2 phase lock gel-heavy, 1 standard). [Phase lock gel tubes are optional for steps 10 and 12. They certainly make it easier to pipette off the upper aqueous sample layer without pipetting phenol or chloroform. However, these layers will separate regardless – and the phase lock gel tubes are currently discontinued (as of October, 2024) – JLB.]

7) After the 90-minute incubation, allow tubes to cool to room temperature

8) Using sterile forceps, remove the swab from each tube and dispose of the swab. In a fume hood (Make sure hood is set to EXTRACTING – NOT RECIRCULATING) add 375 μl of 25:24:1 phenol:chloroform:isoamyl alcohol to the lysate.

9) Agitate gently at room temperature for 5 min by manually inverting

10) Pulse spin down, then move the mixture to a new labeled 1.5 ml phase lock gel-heavy tube, avoiding any undigested solid material

11) Centrifuge for 5 min at 13,000 g to separate the layers.

12) Carefully remove as much of the upper aqueous layer as possible and add to a new 1.5 mL phase lock gel-heavy tube containing 375 μl chloroform. [It is better to leave some of the aqueous layer behind than it is to carry over any of the organic layer or interphase.]

13) Discard the lower organic layer and the old tube into the appropriate hazardous waste.

14) Agitate gently at room temperature for 5 min by manually inverting.

15) Centrifuge for 5 min at 13,000 g to separate the layers.

16) While samples are spinning, add 500 µL of 100% isopropanol to each empty labeled 1.5 ml microcentrifuge tube.

17) Carefully remove the upper aqueous layer and move up to 250 µL to the appropriately labeled 1.5 ml microcentrifuge tube with isopropanol. Discard the lower organic layer and the old tube into the appropriate hazardous waste.

18) If you move < 250 µL of aqueous layer, add elution buffer to bring the volume to 250 µL

19) Add 252.5 µL of the precipitation premix (elution buffer/sodium acetate/glycogen) to each sample normalized to 250 µL.

20) Agitate gently at room temperature for 5 min by manually inverting.

21) Incubate at -20C overnight in a flammable-safe freezer

**Day 4: approximate time for 24 samples – 1-2 hours (TIPS: 200 μl and 1,000 μl)**

22) Centrifuge the tubes at high speed (>14,000g) for 30 min at room temperature.

While tubes are spinning:

- Pre-heat elution buffer in an oven or heat block to 55C
  - 12 samples: 750 μl
  - 24 samples: 1,500 μl
- Make 85% ethanol and chill in a flammable-safe freezer
  - 12 samples: ~5,200 μl ethanol, ~1000 μl water
  - 24 samples: ~10,400 μl ethanol, ~2000 μl water

23) Immediately following centrifugation, carefully remove the liquid from the tube by pouring or pipetting. The DNA will have precipitated into a pellet at the bottom of the tube and may not be visible.

24) To rinse the pellet, gently add 500 μl cold 85% ethanol, gently invert 5x, and centrifuge at 5 min at high speed (>14,000g).

25) Gently decant ethanol using a 200 μl pipette tip to remove what liquid has pooled on the bottom. But be careful not to aspirate the pellet. (Often it is more readily visible after the 85% ethanol wash). Autoclaved toothpicks can be used to blot up drops of ethanol on the side of the tube. All ethanol must be removed from the pellet, as any residual ethanol may inhibit downstream applications.

26) Leave the tube open to dry for 10-20 minutes to continue drying after all residual ethanol has been removed.

27) Re-suspend the pellet in 60 µL of the warmed elution buffer. Allow samples to sit at 4C overnight so the pellet can resuspend.

**Day 5: approximate time for 24 samples – 1 hour (TIPS: 10 μl, 200 μl, and 1,000 μl)**

Before beginning the Day 5 protocol steps:

- Label set of Qubit sample tubes + 2 standard tubes
- If you do not have a pre-mixed working solution, create your own aliquot:
  - 24 samples: 4,776 μl buffer + 24 μl reagent
  - 24 samples + 2 standards: 5,174 μl buffer + 26 μl reagent
- Add 198 μl of Qubit working solution to each sample tube
- Make Qubit standards (10 μl of each standard (1 & 2) in separate tubes + 190 μl Qubit working solution)

28) Add 2 μl of each sample to the corresponding sample tube. Obtain a Qubit value of dsDNA concentration for each sample.

*Section 2. qPCR reaction components and cycling conditions*

Each reaction was composed of:

- 12.5 μL Sensi-Fast Lo-ROX master mix (Meridian Bioscience)
- 1.13 μL of 20 uM ITS1-3 primer (20 µM)
- 1.13 μL of 20 uM 5.8S Chytr primer (20 µM)
- 1.2 μL of Chytr MGB2 probe (5 µM) (FAM labeled)
- 1.0 μL of 100x BSA (100x = 10 mg/mL)
- 0.83 μL of 10x Exo-IPC mix (VIC-labeled)
- 0.17 μL of Exo-IPC DNA
- For museum extracts:
  - 7 μL of template DNA
- For modern extracts:
  - 5 μL of template DNA
  - 2 μL of nuclease-free water
- For Pisces Molecular plasmid Bd standards:
  - 2 μL of plasmid standard
  - 5 μL of nuclease-free water
- For gBlock® Bd standards:
  - 5 μL of gBlock® standard
  - 2 μL of nuclease-free water
- For no-template qPCR controls:
  - 7 μL of nuclease-free water

The thermocycling program was set as follows:

- 2 minutes at 50°C
- 10 minutes at 95 °C
- 50 cycles of 15 seconds at 95 °C
- 1 minute at 60 °C

*Section 3. ddPCR reaction components and cycling conditions*

Each reaction was composed of:

- 10.5 μL 2x ddPCR Supermix for Probes (no dUTP)
- 0.95 μL of ITS1-3 primer (20 µM)
- 0.95 μL of 5.8S Chytr primer (20 µM)
- 1.0 μL of Chytr MGB2 probe (5 µM) (FAM labeled)
- 0.7 μL of 100x BSA (100x = 10 mg/mL)
- 0.83 μL of 10x Exo-IPC mix (VIC-labeled)
- 0.14 μL of Exo-IPC DNA
- 1.0 μL of nuclease-free water
- For museum extracts:
  - 5 μL of template DNA
- For Bd standards:
  - 2 μL of standard
  - 3 μL of nuclease-free water
- For no-template qPCR controls:
  - 5 μL of nuclease-free water

Droplet generation followed standard Bio-Rad protocols.

The thermocycling program was set as follows:

- 2 minutes at 50°C
- 10 minutes at 95 °C
- 50 cycles of 15 seconds at 95 °C
- 1 minute at 60 °C

**Supplementary Tables**

**Table S1:** Summary of all extracted swabs by species, decline-history category, and decade, with summary of extracted control swabs below.

| **Species** | **1940s** | **1970s** | **1980s** | | **2020s** |
| --- | --- | --- | --- | --- | --- |
| *Crossodactylus dispar* | **0** | **14** | Presumed extirpated | | **0** |
| *Cycloramphus boraceiensis* | **1** | **12** | Presumed extirpated | | **0** |
| *Cycloramphus semipalmatus* | **0** | **11** | Presumed extirpated | | **0** |
| *Hylodes asper* | **5** | **2** | Presumed extirpated | | **0** |
| *Hylodes phyllodes* | **3** | **9** | Drastic decline | | **74** |
| *Thoropa taophora* | **10** | **6** | Presumed extirpated | | **0** |
| *Adenomera cantitata* | **1** | **15** | **4** | | **48** |
| *Ischnocnema henselii* | **0** | **31** | **7** | | **154** |
| *Ischnocnema parva* | **0** | **18** | **4** | | **122** |
| *Haddadus binotatus* | **0** | **20** | **6** | | **6** |
| *Scinax hayii* | **0** | **10** | **10** | | **28** |
| *Boana bandeirantes* | **0** | **13** | **20** | | **15** |
| *Rhinella ornata* | **0** | **11** | **4** | | **23** |
| *Rhinella icterica* | **0** | **6** | **2** | | **5** |
| *Dendropsophus minutus* | **1** | **11** | **20** | | **58** |
| *Scinax crospedospilus* | **0** | **10** | **10** | | **5** |
| *Physalaemus cuvieri* | **0** | **10** | **10** | | **1** |
| **Total:** | **21** | **209** | **97** | | **539** |
|  | **Control type** | | | **# extracted** | |
|  | Jar swab control | | | **33** | |
|  | Tube blank extraction control | | | **4** | |

**Table S2:** Evidence supporting decline history inferences for each species.

| **Species** | **Heyer et al. 1988** | **1970s** | **1980s** | **Decline history** |
| --- | --- | --- | --- | --- |
| *Crossodactylus dispar* | extirpated | 14 | 0 | observed-decline |
| *Cycloramphus boraceiensis* | extirpated | 12 | 0 | observed-decline |
| *Cycloramphus semipalmatus* | extirpated | 11 | 0 | observed-decline |
| *Hylodes asper* | extirpated | 2 | 0 | observed-decline |
| *Hylodes phyllodes* | decline | 9 | 0 | observed-decline |
| *Thoropa taophora* | extirpated | 6 | 0 | observed-decline |
| *Adenomera cantitata* | decline | 55 | 10 | observed-decline |
| *Ischnocnema henselii* | decline | 69 | 6 | observed-decline |
| *Ischnocnema parva* | decline | 73 | 5 | observed-decline |
| *Haddadus binotatus* | N/A | 20 | 7 | decline-ambiguous |
| *Scinax hayii* | no decline | 30 | 19 | decline-ambiguous |
| *Boana bandeirantes* | no decline | 118 | 32 | decline-ambiguous |
| *Rhinella ornata* | no decline | 12 | 4 | decline-ambiguous |
| *Rhinella icterica* | no decline | 10 | 16 | no-decline |
| *Dendropsophus minutus* | no decline | 18 | 30 | no-decline |
| *Scinax crospedospilus* | no decline | 11 | 41 | no-decline |
| *Physalaemus cuvieri* | no decline | 21 | 25 | no-decline |

**Table S3:** Bd prevalence and binomial 95% confidence intervals (95% CI) for each species sampled at Boracéia during the 1980s post-decline period.

| **Species** | **Sample size** | **Number infected** | **Bd prevalence (95% CI)** |
| --- | --- | --- | --- |
| Adenomera cantitata | 4 | 0 | **0.000** (0.000, 0.602) |
| Ischnocnema henselii | 7 | 0 | **0.000** (0.000, 0.410) |
| Ischnocnema parva | 4 | 0 | **0.000** (0.000, 0.602) |
| Haddadus binotatus | 6 | 1 | **0.167** (0.004, 0.641) |
| Scinax hayii | 10 | 6 | **0.60** (0.262, 0.878) |
| Boana bandeirantes | 20 | 13 | **0.650** (0.408, 0.846) |
| Rhinella ornata | 4 | 3 | **0.750** (0.194, 0.994) |
| Rhinella icterica | 2 | 1 | **0.50** (0.013, 0.987) |
| Dendropsophus minutus | 20 | 14 | **0.70** (0.457, 0.881) |
| Scinax crospedospilus | 10 | 9 | **0.90** (0.555, 0.997) |
| Physalaemus cuvieri | 10 | 10 | **1.00** (0.692, 1.000) |
| **Overall:** | **97** | **57** | **0.588** (0.483, 0.687) |

**Table S4:** Bd prevalence and binomial 95% confidence intervals (95% CI) for each species sampled at Boracéia during the 2020s.

| **Species** | **Sample size** | **Number infected** | **Bd prevalence (95% CI)** |
| --- | --- | --- | --- |
| Hylodes phyllodes | 74 | 27 | **0.365** (0.256, 0.485) |
| Adenomera cantitata | 48 | 10 | **0.208** (0.105, 0.350) |
| Ischnocnema henselii | 154 | 59 | **0.383** (0.306, 0.465) |
| Ischnocnema parva | 122 | 24 | **0.197** (0.130, 0.278) |
| Haddadus binotatus | 6 | 2 | **0.333** (0.043, 0.777) |
| Scinax hayii | 28 | 4 | **0.148** (0.040, 0.327) |
| Boana bandeirantes | 15 | 7 | **0.467** (0.213, 0.734) |
| Rhinella ornata | 23 | 5 | **0.217** (0.075, 0.437) |
| Rhinella icterica | 5 | 0 | **0.000** (0.000, 0.522) |
| Dendropsophus minutus | 58 | 17 | **0.293** (0.181, 0.427) |
| Scinax crospedospilus | 5 | 3 | **0.600** (0.147, 0.947) |
| Physalaemus cuvieri | 1 | 1 | **1.000** (0.025, 1.000) |
| **Overall:** | **539** | **159** | **0.295** (0.257, 0.335) |

**Table S5:** Proportion of specimens in each decline history category by year in the pre-decline period.

|  | **Proportion of specimens** | | | | |
| --- | --- | --- | --- | --- | --- |
| **Decline history** | **1947** | **1948** | **1975** | **1976** | **1977** |
| Observed-decline | **0.900**  (N = 9) | **1.000**  (N = 11) | **0.632**  (N = 43) | **0.543**  (N = 51) | **0.511**  (N = 24) |
| Decline-ambiguous | NA | NA | **0.279**  (N = 19) | **0.245**  (N = 23) | **0.255**  (N = 12) |
| No-decline | **0.100**  (N = 1) | NA | **0.088**  (N = 6) | **0.213**  (N = 20) | **0.234**  (N = 11) |

**Table S6:** Proportion of specimens in each decline history category by year in the post-decline period.

|  | **Proportion of specimens** | | | | | |
| --- | --- | --- | --- | --- | --- | --- |
| **Decline history** | **1983** | **1984** | **1988** | **2022** | **2023** | **2024** |
| Observed-decline | **0.143**  (N = 7) | **0.269**  (N = 7) | **0.045**  (N = 1) | **1.000**  (N = 80) | **0.565**  (N = 113) | **0.792**  (N = 205) |
| Decline-ambiguous | **0.286**  (N = 14) | **0.423**  (N = 11) | **0.682**  (N = 15) | NA | **0.210**  (N = 42) | **0.116**  (N = 30) |
| No-decline | **0.571**  (N = 28) | **0.308**  (N = 8) | **0.273**  (N = 6) | NA | **0.225**  (N = 45) | **0.093**  (N = 24) |

**Table S7:** Proportion of Bd-positive specimens from each decline history category by year during the post-decline period.

|  | **Bd prevalence (95% CI)** | | | | | |
| --- | --- | --- | --- | --- | --- | --- |
| **Decline history** | **1983** | **1984** | **1988** | **2022** | **2023** | **2024** |
| Observed-decline | **0.000**  (0.000 – 0.410) | **0.000**  (0.000 – 0.410) | **0.000**  (0.000 – 0.975) | **0.288**  (0.192 – 0.399) | **0.389**  (0.299 – 0.486) | **0.259**  (0.200 – 0.324) |
| Decline-ambiguous | **0.429**  (0.177 – 0.711) | **0.364**  (0.109 – 0.692) | **0.867**  (0.595 – 0.983) | NA | **0.262**  (0.139 – 0.420) | **0.233**  (0.099 – 0.423) |
| No-decline | **0.929**  (0.765 – 0.991) | **0.500**  (0.157 – 0.843) | **0.667**  (0.223 – 0.957) | NA | **0.311**  (0.182 – 0.466) | **0.292**  (0.126 – 0.511) |

**Supplementary Figures**

**Figure S1:** Predicted Bd consensus lineage based on combined genotyping results from the nuclear and mitochondrial SNP assays. Boxes are colored based on the lineage associated with each lineage (blue: Bd-GPL, teal: coinfection, green: hybrid, yellow: Bd-Brazil). A black outline indicates that a call can be made using only the mitochondrial assay result.

**Figure S2**: Consensus histopathology results across the 1970s and 1980s. These results integrate pathological diagnostics from both H&E stain and fungal-specific PAS stain. Dark red line represents GAM-modeled predictions for the proportion of samples with detectable Bd sporangia across years with a 95% confidence interval ribbon. Vertical dashed red line represents the observed 1979 Boracéia decline.

***Figure S3****: Bd infection prevalence is reduced and no longer varies by decline history during the 2020s enzootic period. Per-species Bd infection prevalence is shown with 95% binomial confidence intervals. Species are grouped by a priori decline history categories, with category-level prevalence estimates and 95% confidence intervals shown.*
